# Supplementary material for: Sequential conformational transitions and α-helical supercoiling regulate a sensor histidine kinase
Source: Nat Commun. 2017 Aug 18;8:284. doi: 10.1038/s41467-017-00300-5 (PMC5561222; doi:10.1038/s41467-017-00300-5)
Supplement: Supplementary file 1 — Supplementary Information [file 41467_2017_300_MOESM1_ESM.pdf]

Description of Supplementary Files

File name: Supplementary Information
Description: Supplementary figures.

File name: Peer review file

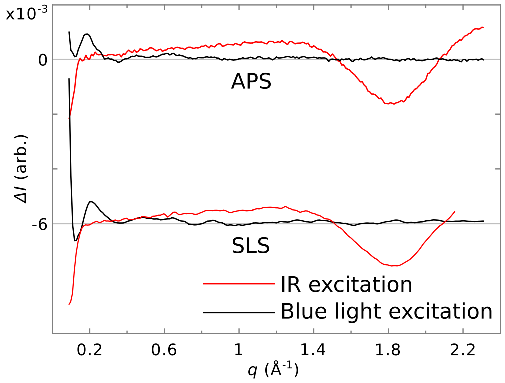

Supplementary Fig. 1: Solvent heating. The difference scattering curves generated by heating the protein solution with an infrared pulse compared to difference scattering curve generated by exciting the chromophore with blue light. For clarity the heat curve has been up scaled. There is essentially no contribution of the heat signal in the blue light excited difference scattering curve. None of the features found in the blue light excitation difference curves can be found in the pure heating curves. Note that the heat curves have not been subtracted from the "Blue light excitation" curves at this stage.

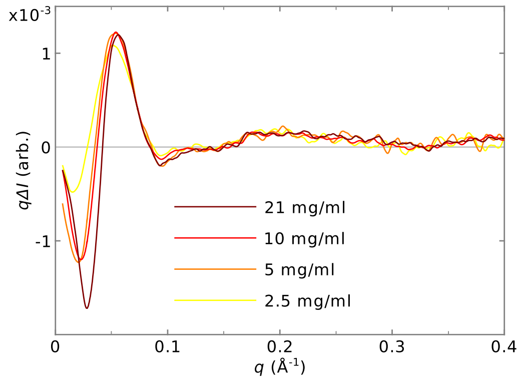

Supplementary Fig. 2: The concentration dependence of the difference scattering curve. The data was collected at cSAXS at the SLS.

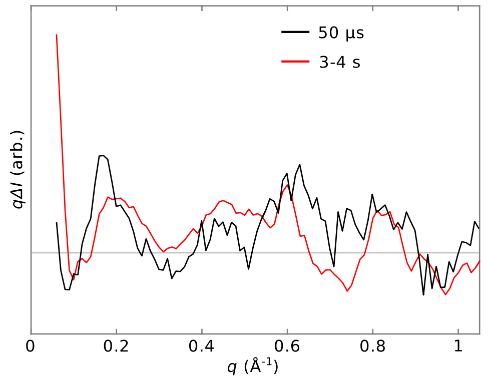

Supplementary Fig. 3: $YF1_{int.}$ and $YF1_{fin.}$ in the raw data. Difference scattering measured at 50 µs and averaged from 3 s to 4 s illustrating the difference between $YF1_{int.}$ and $YF1_{fin.}$. The difference between the two curves also shows that the scattering data is collected to a sufficient level of signal-to-noise.

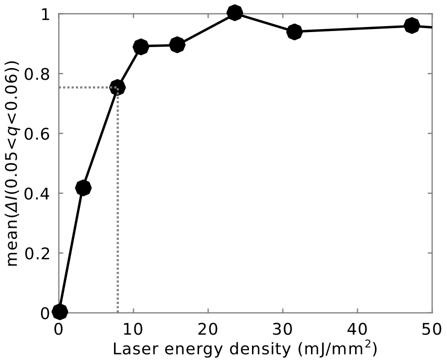

Supplementary Fig. 4: Difference scattering signal amplitude dependence on laser pulse duration. The laser energy (dotted line) at which the main experiment at cSAXS was conducted. The signal is normalized.

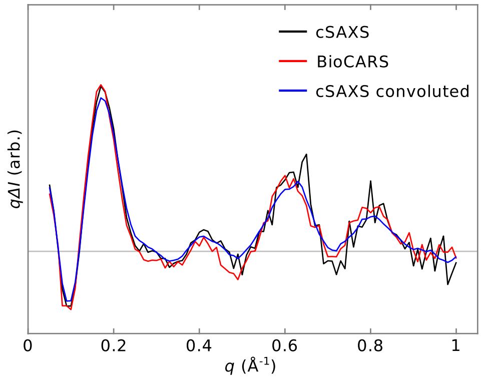

Supplementary Fig. 5: Data reproducibility. Variation between experiments performed at cSAXS and BioCARS and the effect of convoluting the monochromatic scattering curve from cSAXS with the BioCARS undulator spectrum.
